# Stage IVA Esophageal Adenocarcinoma Diagnosed Post-Esophagectomy for End-Stage Achalasia

**DOI:** 10.7759/cureus.89164

**Published:** 2025-07-31

**Authors:** Francisco M Pascual, Charles Cavalaris, Shreya Narayanan, John Jacobs

**Affiliations:** 1 Department of Internal Medicine, University of South Florida Morsani College of Medicine, Tampa, USA; 2 Division of Digestive Disease and Nutrition, Department of Internal Medicine, University of South Florida Morsani College of Medicine, Tampa, USA; 3 Division of Digestive Diseases and Nutrition, Joy McCann Culverhouse Center for Swallowing Disorders, University of South Florida Morsani College of Medicine, Tampa, USA

**Keywords:** esophageal achalasia, esophageal cancer, esophageal disorder, esophageal motility disorder, megaesophagus

## Abstract

Achalasia is a disorder of unknown etiology that disrupts esophageal motility and esophagogastric junction outflow. Many long-term complications are associated with achalasia, including progression to megaesophagus and an increased risk for esophageal cancer. While current guidelines recommend against routine screening for cancer in patients with achalasia, many experts believe that routine endoscopic or radiographic screening at a yet-to-be-determined interval could provide essential data beyond evaluating for cancer. Herein, we present a case of stage IVA esophageal adenocarcinoma diagnosed post-esophagectomy in a patient with end-stage achalasia who underwent irregularly spaced, infrequent endoscopic evaluation.

## Introduction

Achalasia is an esophagogastric junction (EGJ) outflow disorder defined by elevated integrated relaxation pressure and 100% failed peristalsis on high-resolution esophageal manometry (HRM) [1]. Achalasia is categorized into three subtypes: classic achalasia (type I), achalasia with panesophageal pressurization (type II), and spastic achalasia (type III). Current treatment options are not curative but rather offer palliation of symptomology and prevention of complications through interventions such as pneumatic dilation, botulinum toxin injections, and surgical or endoscopic myotomy [2]. End-stage achalasia is diagnosed when esophageal dilation extends beyond 6 cm on timed barium swallow (TBS), with features of significant tortuosity and anatomical distortion [3,4]. Complications of achalasia may include aspiration, malnutrition, megaesophagus, and esophageal cancer. While squamous cell carcinoma is more typically associated with long-standing achalasia, adenocarcinoma may also occur [5]. This report presents the case of a patient diagnosed with megaesophagus and subsequent stage IVA esophageal adenocarcinoma via surgical pathology obtained during elective esophagectomy for therapeutic management of his end-stage achalasia and highlights the lack of definitive surveillance guidelines and the challenges in diagnosing malignancy in patients with megaesophagus.

## Case presentation

**Figure 1:**
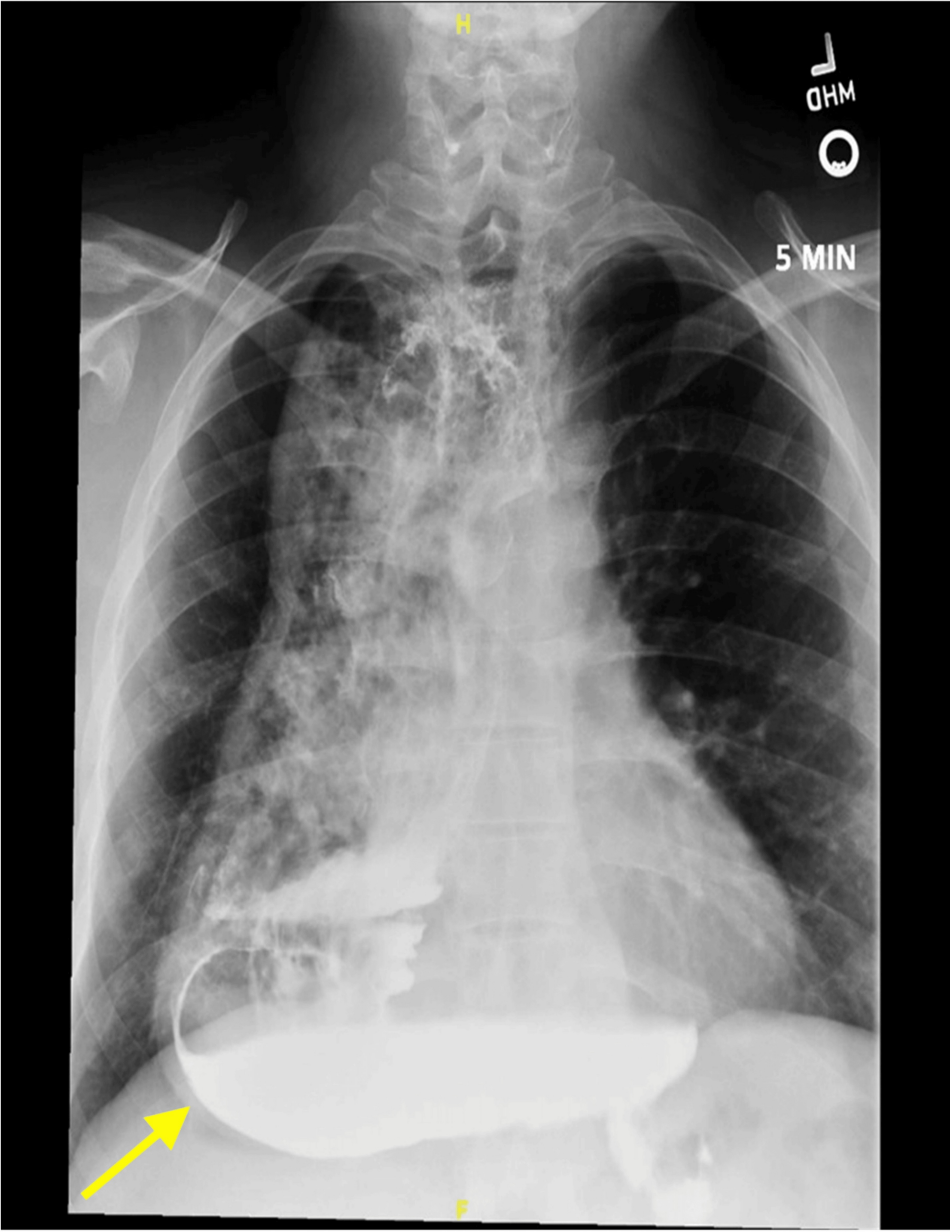
Timed barium swallow at 5 minutes notable for a diffusely distended esophagus with left elbow (yellow arrow) and complete contrast retention.

A 49-year-old man with a past medical history of achalasia was referred to our institution for evaluation of dysphagia. The patient was diagnosed with achalasia at the age of 17 and underwent pneumatic dilation, which was reported to have managed his symptoms for 26 years. At the age of 43, he was hospitalized for chest pain. CT angiography from that admission revealed a significantly dilated esophagus, prompting a laparoscopic Heller myotomy with anterior fundoplication, again with a favorable response. Five years later, the patient was scheduled for bidirectional endoscopy at an outside institution for further evaluation of iron deficiency anemia (IDA). Esophagogastroduodenoscopy (EGD) was attempted, which revealed a dilated esophagus. However, the examination was aborted due to a large volume of retained food in the esophagus. Subsequent upper GI series showed marked esophageal dilatation with significant contrast retention. The patient was thereafter referred to our center for further workup, where HRM demonstrated aperistalsis with 100% incomplete bolus clearance; lower esophageal sphincter pressures could not be obtained as the catheter could not navigate and traverse the EGJ due to abnormal anatomy. Subsequent TBS showed this abnormal anatomy with a massively dilated esophagus with a left elbow and complete contrast retention at 1, 5, and 10 minutes (Figure 1). A diagnosis of end-stage achalasia was made.

The patient’s case was reviewed at a multidisciplinary conference to discuss his left elbow esophageal anatomy, occupying almost half of the thoracic cavity on TBS, and failure of multiple previous therapeutic interventions. Given his clinical context, a consensus recommendation for total esophagectomy was made. At the time of surgery, intraoperative EGD confirmed the presence of megaesophagus with 500 cc of retained food. Esophagectomy was performed without post-operative complications. Histopathology of dissected tissue was obtained, which revealed a diagnosis of pT4a N2 invasive esophageal adenocarcinoma. The patient was referred to oncology, where he underwent adjuvant chemoradiotherapy, followed by immunotherapy. Now, three years post-operative and two years following his last dose of immunotherapy, the patient remains without evidence of disease recurrence.

## Discussion

Preceding our patient’s diagnosis of esophageal adenocarcinoma, he had a longstanding diagnosis of achalasia with relatively infrequent endoscopic evaluation of his esophagus. Interestingly, his disease was subjectively reported as well-controlled for years following a one-time pneumatic dilation and then again following Heller myotomy. Although an EGD was attempted for IDA, in retrospect, an anemia likely due to his underlying malignancy, the procedure could not be completed due to retained food in the esophagus. This large amount of retained food, as well as his megaesophagus, likely prevented endoscopic identification of his malignancy. In addition, his grossly distorted esophageal anatomy likely obscured radiologic identification of an underlying malignancy.

The identification of pT4a N2 invasive esophageal adenocarcinoma via surgical pathology marks a significant finding regarding the utility of therapeutic interventions for esophageal adenocarcinoma. At present, current literature supports treatment for those diagnosed with stage IVA esophageal adenocarcinoma, such as in this case, with neoadjuvant chemotherapy and radiation, followed by surgical resection [6]. The chemoradiotherapy for esophageal cancer followed by surgery study (CROSS) was a groundbreaking study from the Netherlands that compared survival outcomes of neoadjuvant chemoradiotherapy followed by surgery versus surgery alone in patients diagnosed with esophageal cancer and showed overall survival benefit in the former group [6]. A 10-year follow-up study found that the absolute overall survival outcomes related to the original CROSS study demonstrated a persistent survival benefit of 13% in patients who received neoadjuvant chemoradiotherapy and surgery [7].

Currently, no consensus guidelines exist outlining intervals for obtaining serial biopsies, obtaining imaging, initiating endoscopic evaluation post-diagnosis, or defining the frequency of EGD evaluation in patients with achalasia. While current guidelines recommend against routine endoscopic surveillance for carcinoma, many experts believe that developing radiologic or endoscopic surveillance intervals (such as every three years after an established achalasia diagnosis of 10-15 years) may play an essential role in the long-term management of achalasia beyond cancer screening [4]. As HRM has become a widely available tool for diagnosis, recent data support an expected continued rise in the incidence and prevalence of this disease [8]. Given that no reliable biomarkers exist to track the progression of achalasia, clinicians are forced to rely on symptomatology, along with endoscopic and radiographic examination, to guide decision-making on further diagnostic and therapeutic planning.

## Conclusions

This case highlights a key area for growth in the nuanced, long-term management of achalasia. As seen in this case, patient symptoms may not always be reflective of the extent of underlying disease progression, and complications such as megaesophagus may still develop. In this patient, the diagnosis of malignancy was made only upon surgical resection, and pre-operatively, the patient’s megaesophagus, a large volume of retained food, and distorted anatomy likely precluded a diagnosis of malignancy. In addition, while squamous cell carcinoma is more common in achalasia patients, adenocarcinoma can also occur. In this patient, performing routine endoscopy as part of an established surveillance interval may have allowed for the earlier detection of his underlying disease progression. Addressing the current absence of management guidelines involving long-term interval surveillance represents an opportunity for further research and statistical analysis to provide higher-quality care in an attempt to increase the long-term survivability of this patient population.
